# Application of Innovative Ropes from Textile Waste as an Anti-Erosion Measure †

**DOI:** 10.3390/ma14051179

**Published:** 2021-03-03

**Authors:** Giang Nguyen, Joanna Grzybowska-Pietras, Jan Broda

**Affiliations:** 1 Faculty of Civil Engineering, University of Žilina, Univerzitná 8215/1, 010 26 Žilina, Slovakia; 2 Faculty of Materials, Civil and Environmental Engineering, University of Bielsko-Biala, Willowa 2, 43-309 Bielsko-Biala, Poland; jpietras@ath.bielsko.pl (J.G.-P.); jbroda@ath.bielsko.pl (J.B.)

**Keywords:** post-consumer textile waste, wool waste, erosion, anti-erosion measure, Kemafil technology, slope stability, slope stabilization, geotextile tension strength, soil shear strength

## Abstract

Using materials from recycling is a key part of decreasing present-day waste. It is optimal for recycled material to be used in environmental protection. This paper presents the application of geotextile ropes in erosion protection of a slope of a gravel pit. To protect the slope, thick ropes with a diameter of 120 mm made from wool and a mixture of recycled natural and synthetic fibers were used. After 47 months from installation, soil and rope specimens were taken from the slope parts with inclinations 1:1 and 1:1.8, and their physical and mechanical properties were determined. Direct shear tests were applied to determine the soil shear strength parameters in state at sampling and at I_c_ = 0 (unconsolidated and consolidated). Based on the obtained soil shear strength parameters, the loads on the ropes were determined, taking into account also unfavorable hydraulic conditions and compared to rope strength. It was shown that even after 47 months from installation, rope tension strength was higher as tension forces were induced in the ropes in every case. At present, whole slopes in protected sections are stabilized, without rills and gullies.

## 1. Introduction

The current system for producing, distributing, and using clothing operates in an almost completely linear way. Large amounts of nonrenewable resources are extracted to produce clothes that are often used for only a short period, after which the materials are largely lost to landfill or incineration. This linear system leaves economic opportunities untapped, puts pressure on resources, pollutes and degrades the natural environment and its ecosystems, and creates significant negative societal impacts at the local, regional, and global scales [1]. Global material flow analysis shows that less than 1% of material is recycled into the same or similar quality applications; approximately 12% of material is recycled into other, lower-value applications such as insulation material, wiping cloths, or mattress stuffing; approximately 73% of material is landfilled or incinerated [1]. The EU textile industry generates an estimated 16 million tons of waste per year. Much of this waste is thrown into landfills or incinerated, with a high environmental impact and at great cost. Valuable resources held within the waste are also lost [2].

Therefore, research is being carried out to determine ways to utilize textile waste in various sectors, including the building industry. Thus, based on the results of various tests, the authors in [3] stated that their researched textile waste fibers can be incorporated into mortars, thereby replacing sand and cement by volume and adding advantages, such as improved cracking behavior. The authors in [4] investigated the performance of composite materials obtained from the use of fibers derived from recycled textile wastes (100% merino wool), combined with different binders, to be used as building materials with sound absorption and thermal insulation properties. The results confirmed that such materials have similar or better performance than conventional thermal insulating and sound-absorbing materials.

Recycled textiles have better performance than conventional materials, particularly in terms of protecting slopes from erosion. There are some well-known techniques applied to protect a slope from erosion, using geosynthetics such as Husker Fortrac^®^ 3D Geogrid [5] or Presto GEOWEB^®^ [6]. These techniques not only protect a slope from erosion, but also stabilize a slope’s surface. However, the disadvantage of these techniques is that such geosynthetics do not retain water, which can be provided to vegetation on the slope during a drought.

In order to eliminate similar disadvantages, geotextile ropes made from recycled textile were invented a few years ago, where Kemafil technology and various materials (easily available on the local market) were used. The innovative geotextile ropes were meandrically placed on the slope and connected in segments with additional linking chains. The geotextile ropes were successfully used for the stabilization of the slopes in a disused lignite mine, as well as for the protection of drainage slopes, terrace slopes, and road side ditches [7,8,9,10,11,12,13,14].

Since the application of ropes as an anti-erosion measure is quite a new technique, the authors have no knowledge on the literature from other authors evaluating the effectiveness of ropes expressed by quantitative erosion assessment so as to determine the changes in tension force generated in the ropes under various conditions.

Confirmation of the effectiveness of geotextile ropes in terms of their erosion protection of slopes in Lipnik, Międzyrzecze, and Nieboczowy (Poland) can be found in [15]. The effectiveness of the ropes was shown by the actual mean annual soil erosion rate E_A_ and the potential mean annual soil erosion rate E_P_ for two cases: Slopes with ropes and slopes without ropes. The values of E_A_ and E_P_ were calculated by applying the universal soil loss equation (USLE) [16]. The actual mean annual soil erosion rate E_A_ for the slope parts with ropes in all three locations was 0.65, 0.62, and 0.96 t∙ha^−1^∙year^−1^, respectively, classifying these slope parts into class 1—no erosion. The actual mean annual soil erosion rate E_A_ for the slope parts without ropes in all three locations was 14.21, 13.56, and 21.06 t∙ha^−1^∙year^−1^, respectively, classifying these slope parts into class 4—average erosion. The potential mean annual soil erosion rate E_P_ for the slope parts with ropes in all three locations was the same as E_A_, meaning there will be no erosion in the future. However, the potential mean annual soil erosion rate E_P_ for the slope parts without ropes in all three locations was 64.59, 61.62, and 95.73 t∙ha^−1^∙year^−1^, respectively, classifying these slope parts into class 5—strong erosion.

To be successfully applied, geotextile ropes should fulfil the stabilizing function, so no surface slide will occur on the slope. In contrary to the slopes in Lipnik and Międzyrzecze, where the slope inclination was 1:1.5, the slope surface was a plane without slides and the ropes were invisible (buried), the slope in Nieboczowy had various inclinations (from 1:1 to 1:1.8) and the ropes in the steeper part were exposed. The analysis of slope stability presented in a previous paper [17] showed that a slope without geotextile ropes will slide. The shortcomings of the above analysis lie in determination of the physical and mechanical soil properties only on the basis of the soil liquidity index I_L_ in accordance with [18] (not from the tests). The analysis did not include a comparison of the loads induced in the ropes with their tension strength under various conditions. In the present work, the shortcomings of previous analyses were eliminated and the effectiveness of geotextile ropes in slope stabilization was confirmed.

## 2. Materials and Methods

### 2.1. Site Characteristics and Sampling

Investigations were performed in an abandoned gravel pit Nieboczowy, located near Raciborz in southern Poland. The area is located close to the Czech–Polish border in Silesia in the north part of Moravian Gate, with the depression between the Carpathian Mountains in the east and the Sudetes in the west. The site belongs to the floodplain of the Oder river, the second largest river in Poland. The valley is rich in sand and gravel deposits. Abundant deposits are located shallowly under the soil and a thin layer of sand or clay and possess thicknesses locally exceeding 15 m. In the gravel pit in Nieboczowy, after several years of exploitation, the deposits were exhausted and the extraction of gravel was interrupted. As a result of mining, a deep extraction pit with a depth of approximately 10 m was formed. The pit was naturally filled with water to form a small pond. On the banks of the pit, steep unstable slopes prone to local sliding and slipping were generated. In the north-facing bank of the excavation on the surface of native ground, an artificial embankment with a height of approximately 4 m was formed. The embankment erected from overburden material mined during mine operation coupled with the steep slope cut in native ground was especially unstable and endangered by local sliding [17].

For the protection of the slope, geotextile segments formed from thick ropes were applied. The ropes with a diameter of 120 mm were produced by Kemafil technology [19]. The ropes were manufactured from strips of needle-punched nonwoven wool. Wool fibers are of poor quality and obtained from mountain sheep. The fibers possess different diameters and lengths (average thickness 33 µm average length 69 mm). The nonwoven wool had thickness of 5.8 mm, mass per square meter of 406 g∙m^−2^, tension strength of 0.67 kN∙m^−1^, and elongation at break of 40%. The ropes were manufactured also from strips of stitch-bonded nonwoven made from a mixture of recycled natural and synthetic fibers (RNSF rope), obtained by the shredding and carding of post-consumer textile wastes. The raw material is a mixture of synthetic fibers, mostly polyester (approximately 60%) and natural fibers: cotton (approximately 30%) and wool (approximately 10%). The content of the particular fibers is not fixed and varies depending on the amount and kind of waste used. For each portion of the nonwoven, the fibers possess different thickness and strength. The nonwoven had thickness of 3.0 mm, mass per square meter of 265 g∙m^−2^, tension strength of 3.3 kN∙m^−1^ and elongation at break of 35%. Part of the ropes made from recycled fibers was manufactured with the addition of perennial ryegrass (Loliumperenne) seeds (40g per 1 m^2^ of nonwowen).

**Figure 1:**
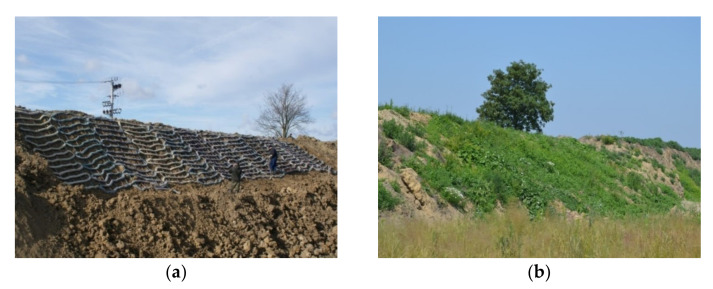
The effectiveness of geotextile ropes as an anti-erosion measure: (**a**) Slope state on 9 February 2016; (**b**) slope state on 23 June 2016 [17].

The ropes were meandrically arranged to form segments with widths of 1.8 m and lengths of 6 m. In order to stabilize the segments, the subsequent turns of the ropes were connected with additional links made from thick polypropylene twine. Geotextile ropes were used to secure a total area of approximately 150 m^2^ in the most threatened part of the slope. The protected slope had a length of 4–6 m and an inclination from 1:1 to 1:1.8. The ropes were anchored in the crown of the slope and fastened at the surface with steel pins. Long “U-shaped” pins made from ribbed bars of diameters ϕ = 8 mm were used. To protect a bigger section of the slope, the subsequent segments of ropes were spread alongside one another. The slope with installed ropes can be seen in the Figure 1a. The effectiveness of installed ropes can be seen in Figure 1b. As we can see in Figure 1a, there is much vegetation, and no signs of erosion exist in the protected slope part [17].

**Figure 2:**
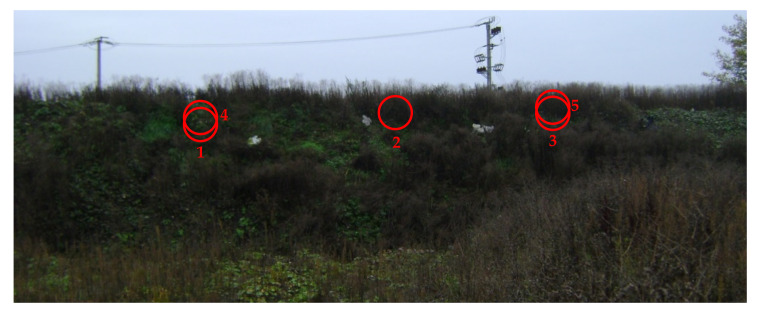
General view of the protected slope in Nieboczowy (numbers mark soil sample locations and state of slope on 22 October 2017; locations 4 and 5 were added on 18 January 2020).

**Figure 3:**
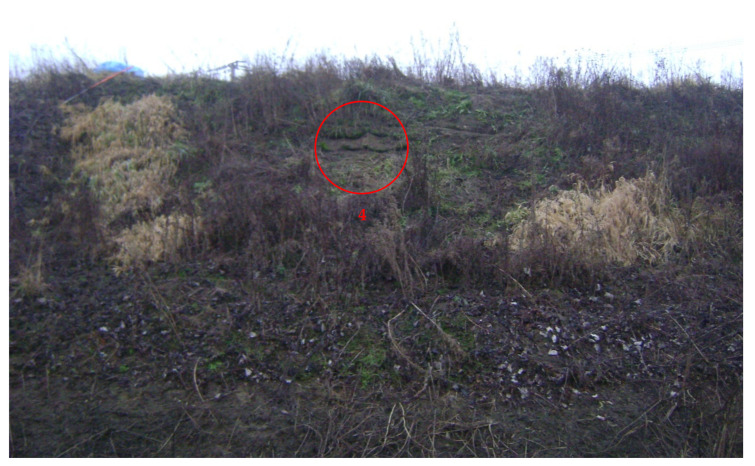
Closer view of location 4 of the protected slope in Nieboczowy (state of slope on 18 January 2020). In the circle: exposed recycled natural and synthetic fibers (RNSF ropes); to the right of the circle: wool rope.

After 20 months after the rope installation, visitation of the protected location took place on 22 October 2017, during which six soil samples were taken from three locations of the protected slope for further analyses. A general view of the protected slope can be seen in Figure 2. Numbers 1, 2, and 3 in Figure 2 mark the soil sample locations, which were chosen from the left, central, and right parts of the slope (horizontally) and in the central part (vertically). Generally, the slope is well protected and covered with rich plants [17]. After 47 months following the rope installation, a second visitation of the protected location took place on 18 January 2020 (47 months after rope installation), during which two soil samples were taken from two locations of the protected slope for further analyses. These locations are marked by the numbers 4 (0.5 m above location 1) and 5 (0.5 m above location 3) in Figure 2. A closer view of location 4 is provided in Figure 3.

**Figure 4:**
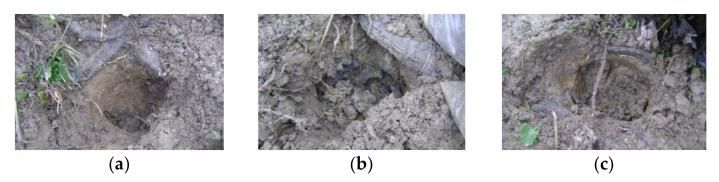
Sampling on 22 October 2017. Detail of the soil sample locations: (**a**) Location 1; (**b**) location 2; (**c**) location 3 [17].

Details of the soil sample locations 1, 2, and 3 with the installed ropes can be seen in Figure 4. It is obvious that the upper soil layer of a thickness of approximately 20 cm (cover layer) seems different to the lower layer. From this reason, two soil samples from every location were taken and analyzed. The specimens were marked based on location (numbers 1, 2, or 3 and letters “a” for the upper layer and “b” for the lower layer); for example, the specimen marked as “1a” was taken from location 1 from the upper layer [17].

**Figure 5:**
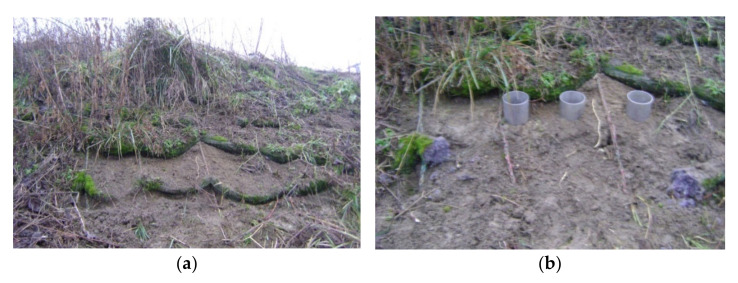
Sampling on 18 January 2020 at location 4. (**a**) The lower rope with three sections (left section, LS; center section, CS; right section, RS). (**b**) The sample cylinders and the cut-off rope for the rope tension strength determination and scanning electron microscopy (SEM).

In order to determine the more precise soil load on the ropes for comparison to the rope tension strength, unlike the previous sampling, soil specimens from the upper layer in locations 4 and 5 were taken as undisturbed. Sampling (soil and rope) at location 4 on 18 January 2020 can be seen in Figure 5. The ropes at location 5 were completely buried in the soil, similarly to locations 1, 2, and 3.

### 2.2. Soil Analyses and Rope Properties

Determination of the soil particle size distribution was carried out in accordance with British Standard BS 1377:1990 Part 2 (the wet sieving method and sedimentation by the hydrometer method) [20]. The soil basic parameters such as water content (w), liquid limits (w_L_), and plastic limits (w_P_) were also determined in accordance with the mentioned standard. Based on the obtained values, soil classification was carried out in accordance with the BS 5930:2015 [21]. Further soil parameters, such as the plasticity index I_P_ and the consistence index I_c_, were calculated by well-known formulas in soil mechanics. 

To analyze the tension force generated in the ropes placed on the slope and its change, it is necessary to know the soil unit weight γ (kN∙m^−3^) and the soil shear strength parameters (angle of internal friction φ (°) and cohesion *c* (kPa)), and their change depending on changes of the consistency index I_c_. These parameters were determined for three states: State on sampling day (18 January 2020), proposed state with I_c_ = 0 (unconsolidated, just after rain), and proposed state with I_c_ = 0 (consolidated, certain time after rain). The soil unit weight was determined based on the soil mass and volume. The soil shear strength parameters were determined by direct shear tests carried out using direct shear apparatus 27-WF2160 (Wykeham Farrance, CONTROLS Group, Milan, Italy) in accordance with the Polish standard [22]. The specimens had sizes of 60 mm × 60 mm × 17 mm; normal stresses were 25, 50, 75, 100, and 125 kPa. The consolidation time was 5 h, and the shear speed was 0.01 mm/min.

Before the installation and at a certain period after the exploitation of the ropes in the soil, including 47 months after rope installation (this research), the mechanical parameters of the nonwovens were measured. The measurements of the tensile strength and elongation at break were carried out in accordance with the Polish standard PN-EN ISO 10319:2010 [23] with a H50K-S Hounsfield tensile machine (Hounsfield Test Equipment Ltd., Redhill, UK). The tested properties of ropes materials are also outlined in [24]. More information on nonwoven properties can be found in [25]. The design tension strength of the ropes was obtained by multiplying the tension strength (kN∙m^−1^) with 1.4 m (width of nonwoven used for production of ropes with diameter 12 cm) and by dividing with 1.1 (proposed reinforcement material factor). The morphology of the fibers was investigated by scanning electron microscopy (SEM). The microscope JEOL JSM 5500 LV (JEOL Ltd., Tokyo, Japan) operating in the backscattered electron mode was used. The observations were carried out for the fibers sputtered with gold in JEOL JFC 1200 ionic sputter. 

### 2.3. Determination of the Soil Load on the Rope

As stated earlier, the authors have no knowledge of the literature from other authors determining changes of tension force generated in the ropes under various conditions. Since the loading mechanism of embankment soil on the geosynthetic crossing piles underpinning the embankment [26] seems to be applicable for Kemafil ropes, we calculated the tension force generated in the ropes based on the mentioned loading mechanism in a further part of this paper.

**Figure 6:**
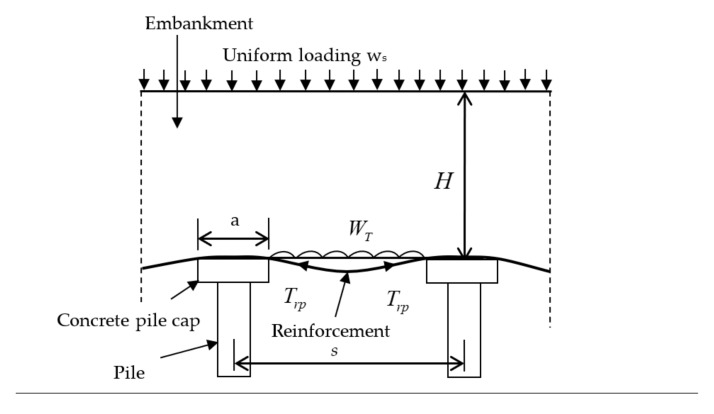
Parameters used in the calculation of $T_{rp}$ [26].

In Figure 6 [26] are the parameters used in the calculation of the tension force *T_rp_* per run meter, generated in reinforcement from vertical loading. For extensible reinforcement, the tension force *T_rp_* per run meter, generated in reinforcement from vertical loading *W_T_,* is:

(1) $$T_{rp}=\frac{W_{T}\left(s-a\right)}{2a}.\sqrt{1+\frac{1}{6\varepsilon }},$$

where (see Figure 6) *T_rp_* is the tension force in the reinforcement (kN), *W_T_* is the vertical loading acting on the reinforcement between two adjacent piles caps (kN), *s* is the distance between the adjacent pile (m) (in our case, the distance between links made from thick polypropylene twine; values of *s* were obtained by measurement), *a* is the size of the pile cap (m) (in our case, we propose 0.05 cm), and *ε* is the strain in the reinforcement (-), which were calculated based on the real rope length and distances *s*.

The above equation has two unknowns: *T_rp_* and *ε*. For *T_rp_*, the unknown can be solved when the maximal allowed deformation of the reinforcement and deformation characteristics of reinforcements at various loadings (loading deformation characteristics) are taken into account. The mentioned equation for *T_rp_* is suitable for these reinforcements, which can undergo deformation during loading, meaning extensible reinforcements (e.g., polymeric). In this case, the abovementioned calculated real values of *ε* were applied.

**Figure 7:**
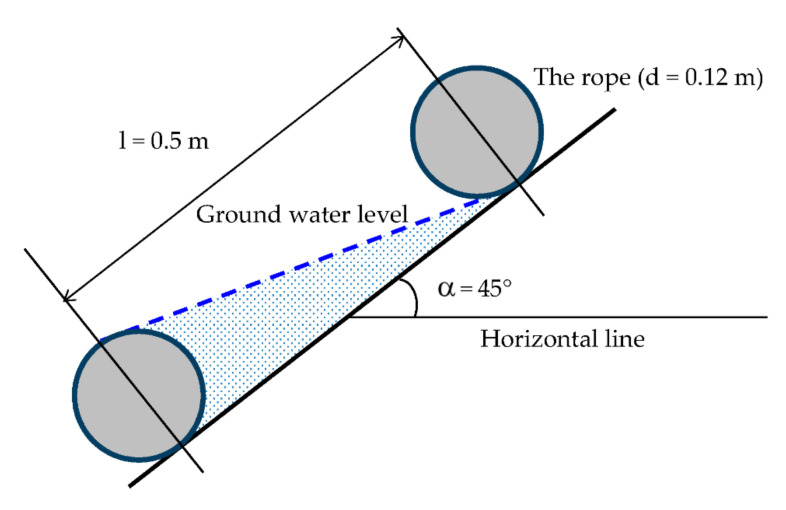
Schema for the calculation of the load acting on the rope at location 4.

The load *W_T_* acting on the rope at location 4 was calculated as the difference between active force (the tangent component of the soil weight, the force from flowing water, and the soil pressure at rest) and passive force (friction and cohesion force on slide surface) using well-known formulas in geotechnical engineering (see Figure 7).

**Figure 8:**
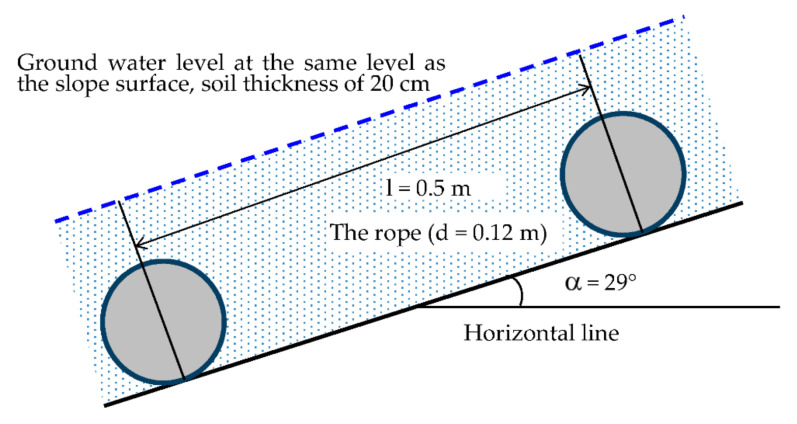
Schema for calculation of load acting on rope at location No. 5 (ropes are buried in soil).

The load *W_T_* acting on rope at location 5 was calculated as the difference between the active force: The tangent component of the soil weight, the force from flowing water, and the passive force (friction and cohesion force on slide surface) using well-known formulas in geotechnical engineering (see Figure 8).

## 3. Results and Discussion 

### 3.1. Soil and Rope Properties

**Figure 9:**
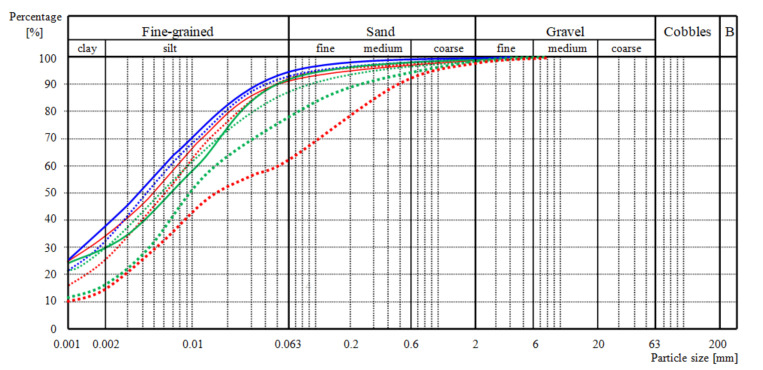
Grain size distribution diagram of the soils. Upper six curves (six samples taken on 22 October 2017): Thin red dashed line and solid line: Location 1, top and down sample; thin blue dashed line and solid line: Location 2, top and down sample; thin green dashed line and solid line: Location 3, top and down sample [17]. Lower two curves (two samples taken on 18 January 2020): The lowest thick red dashed line: Location 4, top sample, approximately 0.5 m from location 1; the lowest thick green dashed line: Location 5, top sample, approximately 0.5 m from location 3).

Grain size distribution diagrams of the soils in the protected section can be seen in Figure 9. The soil classifications and properties are given in Table 1. The grain size distribution diagram of the soils taken from three places on 22 October 2017 are close to one another and the classifications of the soils are very similar (CH—clay of high plasticity and CI—clay of intermediate plasticity—even the distance between locations 1 and 3 was approximately 14.5 m. Only sample number 3b (location 3, lower layer) was classified as CI. However, its liquid limit (w_L_ = 48.9%) was very close to 50%, which is the limit value between CI and CH (see Table 1). For this reason, we can also propose that soils at locations 4 and 5, which were located only approximately 0.5 m above locations 1 and 3, can be classified as soils at locations 1 and 3. The large amount of silty fraction in the soils (from 55.9 up to 63.3%) shows their sensibility to erosion. For the soils from the upper layers (samples 1a, 2a, and 3a), the water content was higher (from 32.8% to 37.9%). This may be evidence that ropes possess ability to hold water and to provide it to growing vegetation. Consequently, the higher water content of the upper layers provided lower consistency indexes (0.55, 0.73, and 0.62). A higher water content is favorable for vegetation growth, but not for slope stability. However, thanks to the installed ropes, the slope remained stable during the visitation [17].

Approximately 27 months later (on 18 January 2020), the grain size distribution diagrams changed. At critical location 4 (approximately 0.5 m from location 1; see Figure 5), where the slope had a general inclination of 1:1 with a step caused by exposed rope; the change was significant, the soil now being classified as CS (sandy clay) with a decreased amount of clayey and silty fraction and an increased amount of sandy and gravelly fraction (compare the thin and thick red dashed lines in Figure 9 and the data for samples 1a and 4a in Table 1). There was also a change in the grain size distribution diagram of the soil from location 5, but it was not so significant (compare the thin and thick green dashed lines in Figure 9 and the data for samples 3a and 5a in Table 1). The amount of clayey fraction also decreased and the amount of sandy and gravelly fraction also increased as in the first case, but in this case, the amount of silty fraction did not decrease, but rather increased a little). We propose that the smaller change in comparison to the first case (soils at locations 1 and 4) was caused by the smaller slope inclination (1:1.8) without a step; furthermore, the rope was buried in the soil (no step caused by exposed rope). The change in grain size distribution and likely also further factors, such as alternate freezing and thawing and vegetation, also caused changes in W_L_ and Wp (compare data in Table 1) and the soil mechanical properties.

**Figure 10:**
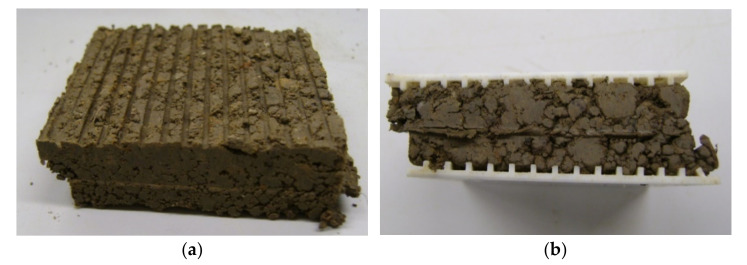
Soil samples after shearing (state of sampling day). (**a**) Sample from location 4, after shearing at a normal stress of 100 kPa; (**b**) sample from location 5, after shearing at a normal stress of 50 kPa.

The results of the direct shear tests of the soils taken on 18 January 2020 are outlined in Table 2 and Table 3. It is worth noting that the angles of the internal friction of both soils (CS and MH—silt of high plasticity at the state of sampling day with a water content of w = 25.9 and 30.7% (I_c_ = 0.83 and 0.93, respectively) were smaller than the angles of the internal friction of both soils at a water content of w = 43.8 and 52.3% (I_c_ = 0 in both cases). In both cases, the cohesions were larger than the cohesions of the consolidated soils, but smaller than the cohesions of the unconsolidated soils. This fact can be explained by the structure of the soils at the state of sampling day, as can be seen in Figure 10, which caused smaller unit weights, as well as smaller contact areas of the soil particles at the shear surface compared to the shear box cross-section. The prepared specimens with a water content equal to the liquid limit had higher unit weights and larger contact areas of the soil particles at the shear surface. The obtained values of the soil’s unit weight and shear strength parameters were applied in the calculation of the loads on the rope. 

**Figure 11:**
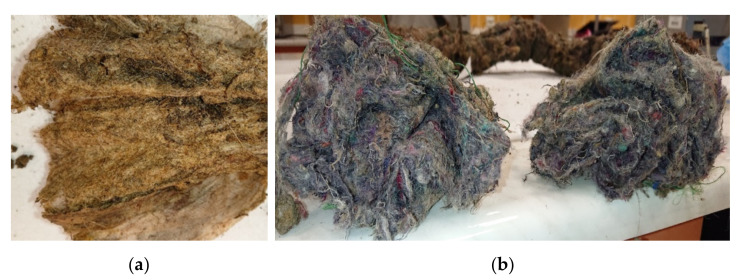
View of the ropes 47 months after installation. (**a**) Wool rope; (**b**) recycled natural and synthetic fiber (RNSF) ropes (left: From location 4; right: From location 5).

Figure 11 presents ropes after 47 months from installation. For ropes manufactured from wool, the symptoms of advanced biological damage are clearly visible. The observed changes of the natural original wool color into yellow and brown stains are the result of a far-reaching wool keratin biodegradation. In contrast to wool, for the RNSF ropes no visible signs are observed. For both parts of the ropes (outer and inner layer) the color of the nonwoven is unchanged and no other changes are visible.

**Figure 12:**
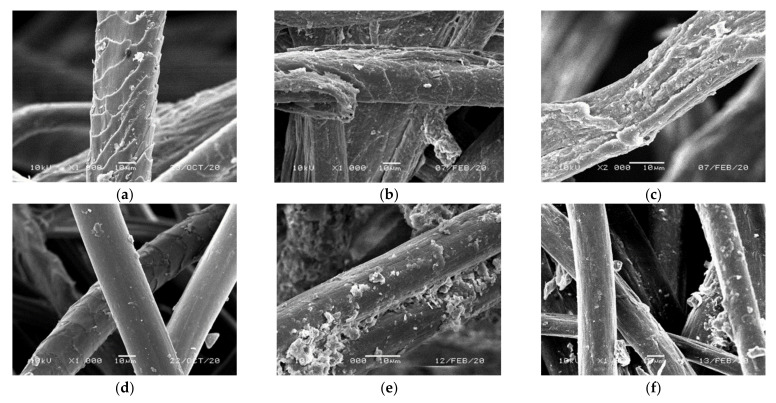
Changes in the morphology of the fibers of the rope depending on the material type. (**a**) Original wool fiber; (**b**) wool fiber from the wool rope surface 47 months after installation; (**c**) wool fiber from the wool rope core 47 months after installation; (**d**) original recycled natural and synthetic fibers; (**e**) recycled natural and synthetic fiber from the RNSF rope surface 47 months after installation; (**f**) recycled natural and synthetic fiber from the RNSF rope core 47 months after installation.

On the SEM images of wool (Figure 12a–c), biodegradation occurring during the exploitation of ropes is confirmed. In the initial step of the process, cuticle cells, forming on the fiber surface characteristic scales, are firstly destroyed (Figure 12a,b). After 47 months of placement in soil, the cuticle cells are completely destroyed and further biodegradation causes the gradual destruction of the deeper placed cortical cells. The destruction leads to fibrils separation, which is followed by the disintegration of the fiber structure (Figure 12c).

For RNSF ropes taken 47 months after installation on the slope no significant signs of biodegradation are observed on the SEM images (Figure 12d–f). For fibers taken from the cover layer of the ropes, significant amount of soil particles attached to the fiber surface is visible (Figure 12e). In addition, slight mechanical damage of the fiber surface resulting from a movement of soil particles is observed. Similarly, on the fibers taken from rope core, some subtle changes of the mechanical origin are noticeable (Figure 12f).

### 3.2. Soil Load on the Rope

The measured mechanical properties of the rope materials are introduced in Table 4 and Table 5 (according to [24,25] and this research). As we can see, the tension strength of wool reduced by 0.07 kN∙m^−1^ (10.4%) after 12 months (no defined detail location in [24]) and was practically null after 47 months (degraded, not tested) in both locations 4 and 5. The tension strength of the RNSF rope at location 4 (not buried, exposed to climate variation) reduced by 0.3 kN∙m^−1^ (9.09%) after 47 months. After 47 months, the tension strength of the RNSF rope at location 5 (completely buried in the soil) reduced by 0.1 kN∙m^−1^ (3.03%) only. We can propose that the different reductions in rope tension strength were caused by different exposures to climate variation.

The design tension forces in the ropes placed on the slope part with inclinations of 1:1 (location 4) and 1:1.8 (location 5) are introduced in Table 6 and Table 7. As we can see, at location 4, tension forces were generated in the ropes in all soil tested states. The tension forces were largest in the right rope section. Concerning the soil state and the related load in the rope, the largest tension forces occurred when the soil had I_c_ = 0.00 and was unconsolidated (proposed just after rain). The second worst state was when the soil had I_c_ = 0.00 and was consolidated (certain period after rain). The tension forces were smallest when the soil had I_c_ = 0.83 (state on sampling day). For all cases, the tension forces generated in the ropes were smaller than the design tension strength of the rope, so no rope failures or slide occurred. Thus, in this case, the rope not only restrained water and provided it to plants during the dry period, but also restrained the soil so that no slide occurred.

Based on the slope at location 5 having an inclination of 1:1.8 (slope angle of approximately 29°), there was no load on the rope (the negative values of the W_Tj_ in Table 7 only illustrate the differences between active and passive forces in the stability analysis). This means that the slope is stable without rope. Thus, in this case, the rope needs to not only restrain soil, but also to provide it to plants during the dry period.

Concerning rope behavior on site, based also on our observation in further locations (Lipnik, Międzyrzecze), where ropes are successfully applied and where slope inclination is 1:1.5, we can state that the ropes are exposed only in this location in the part of the slope with slope inclination larger than 1:1.3. At the present, no exposal of rope can be observed in the slope part flatter than 1:1.3. The first exposal of the rope was observed on 5 May 2016 (about 3 months after the installation) and the ropes have stayed exposed all the time to the present. The cause of the exposal of the rope was slide of soil due to the large slope inclination and also erosion. 

The values of the strain of the ropes are introduced in the Table 6 and Table 7 which are smaller than the values of elongation at break in the Table 5. Comparing values of the rope strain between sections of the rope in location 4 (exposed rope) we can see that there are large differences in the rope strain, e.g., between left section (28%) and center section (13%). These large differences can be caused by various loading conditions during the exploitation. Comparing values of rope strain between sections of the rope in location 5 (rope completely buried in the soil), the differences are smaller. The strains of the rope in location 4 are larger than the strains of the rope in location 5 since load acting on the rope in location 4 is larger than the one acting on the rope in location 5.

The larger strain of rope causes smaller tension forces induced in the rope and vice versa. The values of the tension forces induced in the rope for proposed small strain (e.g., 5%) are introduced in the round brackets in the Table 6. As we can see, proposed induced tension forces are much smaller than rope tension strength. Further research on this issue can contribute to the optimization of rope design.

## 4. Conclusions

The erosion of the slope in Nieboczowy was effectively restricted by geotextile ropes. The central part of the slope (with ropes) was stable, while the left and right parts of the slope (without ropes) were severely affected by erosion.

The grain size distribution of the soils changed significantly after approximately 27 months. Fine particles caused less change in soil classification.

The wool rope was practically degraded and had no tension strength, while the rope made from recycled textile waste retained high tension strength, even after 47 months following installation.

Various soil states caused various loads on the rope. In this case, the rope made from recycled textile waste had sufficient strength to restrain soil, even on the steepest slope part with an inclination of 1:1.

Thus, ropes made from recycled textile waste can be used under various geotechnical conditions. For further applications, the design of ropes and their properties should be optimized using the results of additional laboratory and in situ tests.

## Figures and Tables

**Table 1:** Soil properties on 02 October 2017 (samples 1a–3b) [17] and 18 January 2020 (samples 4a and 5a).

Taken On	2 October 2017						18 January 2020	
Sample No.	1a	1b	2a	2b	3a	3b	4a	5a
Location	No. 1 (upper)	No. 1 (lower)	No. 2 (upper)	No. 2 (lower)	No. 3 (upper)	No. 3 (lower)	No. 4 (upper)	No. 5 (upper)
Graphical presentation	Red dashed line (thin)	Red solid line	Blue dashed line	Blue solid line	Green dashed line (thin)	Green solid line	Red dashed line (thick)	Green dashed line (thick)
Soil classification by [21]	CH	CH	CH	CH	CH	CI	CS	MH
Clayey fraction amount (%)	28.7	34.9	32.1	35.1	30.7	30.9	16.5	16.8
Silty fraction amount (%)	63.3	55.9	61.3	61.4	56.7	62.9	45.5	61.2
Sandy fraction amount (%)	7.8	9.0	6.5	3.5	12.4	6.2	36.6	21.0
Gravelly fraction amount (%)	0.2	0.2	0.1	0.0	0.2	0.0	1.4	1.0
Water content (w) (%)	37.9	26.7	32.9	28.5	32.8	24.9	25.9	30.7
Plastic limit w_P_ (%)	26.2	19.6	24.4	20.8	20.6	19.1	22.4	29.2
Liquid limit w_L_ (%)	52.5	51.9	56.9	59.3	53.1	48.9	43.8	52.3
Plasticity index I_P_ (%)	26.3	32.3	32.5	38.5	32.5	29.7	21.4	23.1
Consistency index I_C_ (-)	0.55	0.78	0.73	0.80	0.62	0.80	0.83	0.93

Notes: CH: clay of high plasticity; CI: clay of intermediate plasticity; CS: sandy clay; MH: silt of high plasticity.

**Table 2:** Water content, unit weight, and shear strength parameters of soil sample 4.

Sample 4a	Normal Stress (kPa)	Shear Stress (kPa)	φ (°)	c (kPa)	R-Squared Value
CS (state on 18 January 2020; w = 25.9%; I_c_ = 0.83; γ = 16.4 kN∙m^−3^)	25	16.5	20.6	6.0	0.9911
	50	24.7			
	75	33.2			
	100	42.1			
	125	54.8			
CS (w = w_L_ = 43.8%; I_c_ = 0; unconsolidated; γ = 18.7 kN∙m^−3^)	25	23.9	25.5	9.4	0.9821
	50	30.8			
	75	42.6			
	100	59.7			
	125	69.1			
CS (w = w_L_ = 43.8%; I_c_ = 0; consolidated; γ = 18.7 kN∙m^−3^)	25	19.6	30.5	4.1	0.9984
	50	33.1			
	75	48.1			
	100	62.0			
	125	79.0			

**Table 3:** Water content, unit weight, and shear strength parameters of soil sample 5.

Sample 5a	Normal Stress (kPa)	Shear Stress (kPa)	φ (°)	c (kPa)	R-Squared Value
MH (state on 18 January 2020; w = 30.7%; I_c_ = 0.93; γ = 15.7 kN∙m^−3^)	25	15.7	21.1	6.0	0.9896
	50	26.4			
	75	34.7			
	100	47.7			
	125	53.3			
MH (w = w_L_ = 52.3%; I_c_ = 0; unconsolidated; γ = 18.4 kN∙m^−3^)	25	18.2	24.8	6.7	0.9866
	50	31.8			
	75	37.8			
	100	53.8			
	125	65.0			
MH (w = w_L_ = 52.3%; I_c_ = 0; consolidated; γ = 18.4 kN∙m^−3^)	25	19.0	27.5	5.4	0.9926
	50	32.8			
	75	41.5			
	100	58.3			
	125	71.5			

**Table 4:** Properties of the textile waste rope materials according to [24,25] (after 0, 6, and 12 months) and this research (location 4).

Nonwoven Materials	Months	Tension Strength (kN∙m^−1^)	Elongation at Break (%)	Design Tension Strength of Ropes T_D(ULS)_ (Nonwoven Width 1.4 m (kN)
Wool	0	0.67	40	0.853
	6	0.62	33	0.789
	12	0.60	31	0.764
	47 (January 2020)	Degraded, not tested		
Recycled textile wastes	0	3.3	35	4.200
	6	3.3	33	4.200
	12	3.3	32	4.200
	47 (January 2020)	3.0	23	3.818

**Table 5:** Properties of the textile waste rope materials according to [24,25] (after 0, 6, and 12 months) and this research (location 5).

Nonwoven Materials	Months	Tension Strength (kN∙m^−1^)	Elongation at Break (%)	Design Tension Strength of Ropes T_D(ULS)_ (Nonwoven Width 1.4 m (kN)
Wool	0	0.67	40	0.853
	6	0.62	33	0.789
	12	0.60	31	0.764
	47 (January 2020)	Degraded, not tested		
Recycled textile wastes	0	3.3	35	4.200
	6	3.3	33	4.200
	12	3.3	32	4.200
	47 (January 2020)	3.2	27	4.073

**Table 6:** Design tension force in the ropes placed on the slope part with an inclination of 1:1 in comparison to the rope design. tension strength (location 4).

Parameters		Distance between Links Made from Thick Polypropylene Twine (m) and Rope Strain (%)		
		0.39 (ε = 28%) (Left Section)	0.46 (ε = 13%) (Center Section)	0.48 (ε = 17%) (Right Section)
I_c_ = 0.83 φ = 20.6° c = 6.0 kPa	W_Tj_ (kN)	0.077	0.091	0.094
	T_j_ (kN)	0.329 (0.543)	0.560 (0.772)	0.574 (0.845)
	T_D_ (kN)	3.818	3.818	3.818
I_c_ = 0.00 (unconsolidated) φ = 25.5° c = 9.4 kPa	W_Tj_ (kN)	0.085	0.100	0.104
	T_j_ (kN)	0.363 (0.598)	0.617 (0.851)	0.633 (0.932)
	T_D_ (kN)	3.818	3.818	3.818
I_c_ = 0.00 (consolidated) φ = 30.5° c = 4.1 kPa	W_Tj_ (kN)	0.082	0.096	0.100
	T_j_ (kN)	0.350 (0.578)	0.596 (0.822)	0.611 (0.899)
	T_D_ (kN)	3.818	3.818	3.818

Notes: Rope diameter, d = 12 cm; cover soil thickness, 12 cm; W_T_, load on rope; T_j_, design tension force in rope (the values in the round brackets are applied for ε = 5%); T_D_, design tension strength of rope; ε, rope strain (calculated as a ratio between the real rope length and the distance between the links).

**Table 7:** Design tension force in the ropes placed on the slope part of an inclination of 1:1.8 in comparison to the rope design. Tension strength (location 5).

Parameters		Distance between Links Made from Thick Polypropylene Twine (m) and Rope Strain (%)		
		0.37 (ε = 12%) (Left Section)	0.53 (ε = 10%) (Center Section)	0.43 (ε = 5%) (Right Section)
I_c_ = 0.93 φ = 21.1° c = 6.0 kPa	W_Tj_ (kN)	−0.407	−0.583	−0.473
	T_j_ (kN)	No force	No force	No force
	T_D_ (kN)	4.073	4.073	4.073
I_c_ = 0.00 (unconsolidated) φ = 24.8° c = 6.7 kPa	W_Tj_ (kN)	−0.527	−0.755	−0.612
	T_j_ (kN)	No force	No force	No force
	T_D_ (kN)	4.073	4.073	4.073
I_c_ = 0.00 (consolidated) φ = 27.5° c = 5.4 kPa	W_Tj_ (kN)	−0.399	−0.572	−0.464
	T_j_ (kN)	No force	No force	No force
	T_D_ (kN)	4.073	4.073	4.073

Note: Rope diameter, d = 12 cm; cover soil thickness, 20 cm; W_T_, load on rope; T_j_, design tension force in rope; T_D_, design tension strength of rope; **ε**, rope strain (calculated as a ratio between the real rope length and the distance between the links).

## Data Availability

Data sharing is not applicable to this article.
